# Intermittent fasting and time-restricted eating role in dietary interventions and precision nutrition

**DOI:** 10.3389/fpubh.2022.1017254

**Published:** 2022-10-28

**Authors:** Ghada A. Soliman

**Affiliations:** Department of Environmental, Occupational, and Geospatial Health Sciences, CUNY Graduate School of Public Health and Health Policy, The City University of New York, New York, NY, United States

**Keywords:** intermittent fasting (IF), time-restricted eating (TRE), fasting-mimicking diets (FMD), precision nutrition, alternative day modified fasting (ADMF)

## Abstract

Intermittent fasting (IF), time-restricted eating (TRE) and fasting-mimicking diets (FMD) are gaining popularity as weight loss programs. As such, the timing and frequency of meals have been recognized as essential contributors to improving cardiometabolic health and a role as adjuvant therapy in cancer. Randomized controlled trials suggested that the weight loss associated with IF is due to a reduced energy intake due to time restriction. Although the supervised TRE clinical trials documented the dietary caloric intake, many free-living studies focused on the timing of meals without a complete characterization of the dietary intake, caloric density, or macronutrient composition. It is possible that both caloric-restriction diets and time-restriction protocols could work synergistically or additively to improve metabolic health outcomes. Like personalized medicine, achieving precision nutrition mandates the provision of the right nutrients to the right patient at the right time. To accomplish this goal, future studies need to evaluate the benefits of IF and TRE. Randomized controlled trials were conducted in different populations, ethnic groups, ages, geographic distribution, physical activity levels, body composition and in patients with obesity, diabetes, and cardiovascular diseases. Also, it is crucial to analyze the dietary composition and caloric density as related to circadian rhythm and timing of meals. It is conceivable that IF and TRE may contribute to precision nutrition strategies to achieve optimal health. However, more research is needed to evaluate IF and TRE effects on health outcomes and any side effects.

## Introduction

Fasting is defined as voluntary abstinence from food and drinks for a specific period of time (1). Metabolically, fasting could be categorized into a post-absorptive state, fasting and starvation. In the post-absorptive state, where no glucose or other nutrients are ingested, glycogenolysis, which is the breakdown of glycogen in the liver, provides glucose to the blood and tissues. Approximately after 18–24 h. Without food, the hepatic glycogen is depleted, and gluconeogenesis, the synthesis of glucose from non-carbohydrates sources such as fat, lactate and eventually amino acids, occurs, leading to the *de novo* synthesis of glucose for energy production. It has been suggested that fasting has a multitude of health benefits [reviewed in (1, 2)]. Therefore, various forms of intermittent fasting and time-restricted eating have been developed over the last decade.

This perspective summarizes some of the current protocols in fast-mimicking diets. Intermittent fasting (IF) and time-restricted eating (TRE) refer to predetermined timing in pausing or abstaining from eating followed by ingestion of food (3, 4). In this approach, macronutrients providing calories are consumed in a time-specific window. Most free-living clinical studies do not restrict caloric consumption at the time-restricted window, which is considered an advantage to improving adherence as no calorie count is needed. On the other hand, traditional comprehensive weight management programs encompass caloric restriction, either as a low-calorie diet (LCD) or a very low-calorie diet (VLCD) under particular circumstances (5, 6), in addition to physical activity and behavior modification (7).

Furthermore, if co-morbidities exist or at a specific Body Mass Index (BMI), pharmacological drugs and weight loss surgery may be added to the low-calorie diet, physical activity, and behavior modification protocols (8–11). However, the timing and frequency of meals have been recognized as essential contributors to improving cardiometabolic health, weight loss, and other health benefits, which will be discussed in the following paragraphs.

## Intermittent fasting

**Figure 1:**
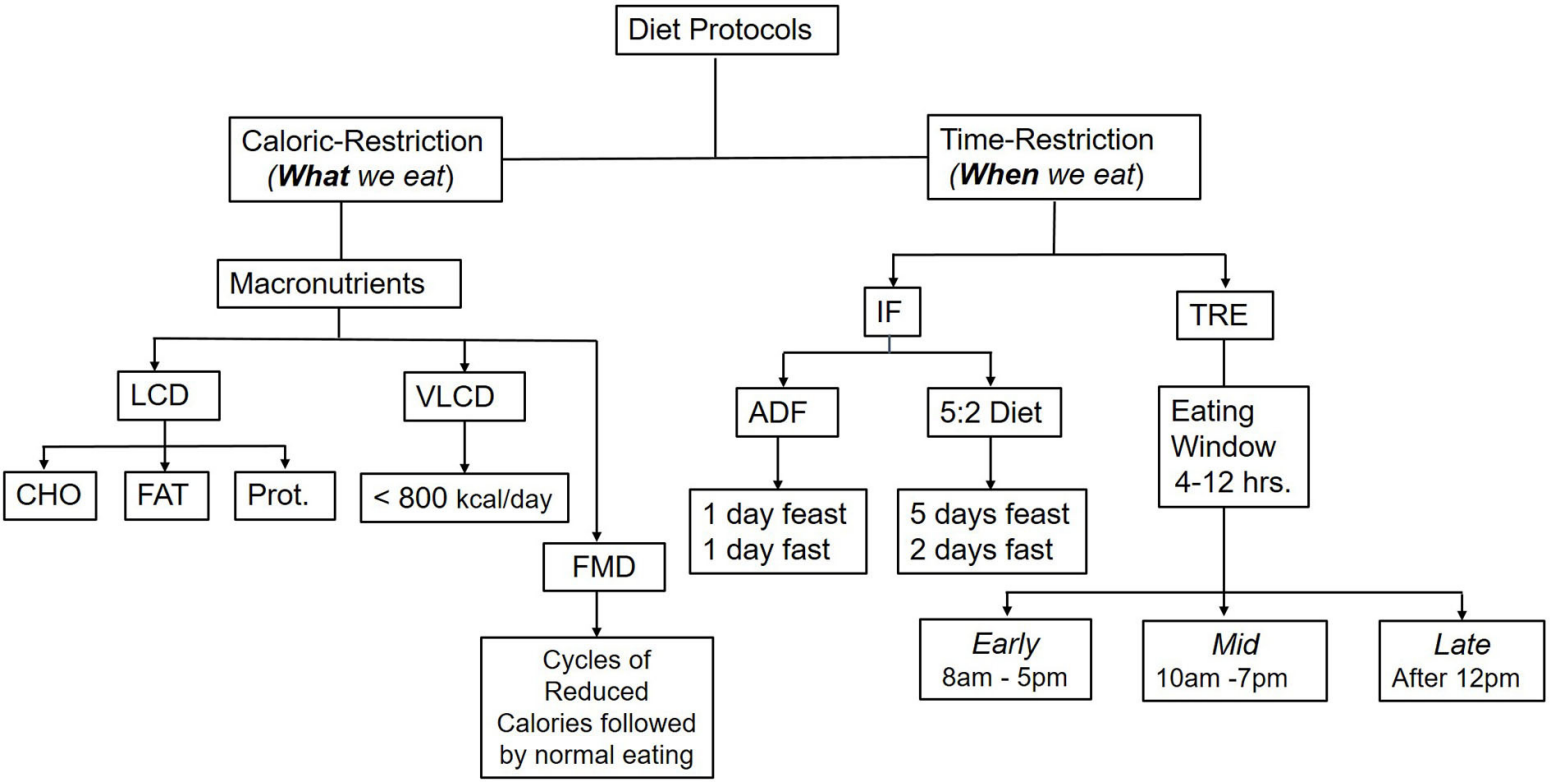
Common caloric-restriction and time-restriction diet protocols. Comprehensive weight management programs include a low-calorie diet (LCD), physical activity and behavior modifications. In addition, pharmacological drugs or weight loss (bariatric surgery) may be added if co-morbidities exist or at a certain high BMI. LCD could be either a low carbohydrate (CHO) diet, low fat (FAT), or under specific circumstances, low protein (Prot.). A very low-calorie diet (VLCD), which provides only 800 kcal/day, is used only with medical supervision. Time-restricted diets include Intermittent fasting (IF), Time-restricted eating (TRE), and Fasting-mimicking diets (FMN). TRE could be either at the earlier part of the day, starting from 8 AM to 5 PM, or midday with delayed breakfast from 10 AM to 7 PM, or late from 12 PM−8 PM or a variation of these times as a personal preference.

Refers to a dietary strategy that alternates feeding days with fasting days (12). The most common methods are alternating days of fasting (ADF) with one-day feasting and 1-day fasting or a 5:2 diet with 5 days of feasting and 2 days of fasting (either two consecutive fasting days or two separate days within the week). IF protocols do not restrict feeding during the feasting days, where people are allowed to eat normally or *ad libitum* and then abstain from eating for one or 2 days (Figure 1); however, some protocols allow for up to 500 kcal per day on fasting days (13).

## Time-restricted eating

Refers to restricting the time when meals are consumed within 24 h. Most TRE studies to date ranged from 4–12 h of eating window without caloric restriction. Some investigators consider TRE a chrono-nutritional strategy (14, 15). The flexibility of the TRE protocols allows for maintaining individual eating pattern preferences, which may facilitate adherence to and compliance with the diet protocol. In addition, alternating feeding and fasting cycles following the circadian rhythm, i.e., eating during the active phase and fasting during the resting phase, may also have a favorable effect on nutrient metabolism, hormonal regulation and physiological processes and thereby may improve cardiometabolic health.

## Fasting-mimicking diets

FMD differs from the IF in that it allows for reduced calorie intake instead of complete abstinence of food during the fasting period FMD constitutes periodic cycles of consecutive days consuming a reduced-calorie diet followed by eating *ad libitum* (15, 16). FMD could be a plant-based diet, low in protein and sugar content. FMD has emerged as another dietary modification that could be beneficial in cardiometabolic diseases and weight loss programs (16–18), as well as an adjuvant intervention in addition to cancer therapy (19–22). Since FMD does not include restricting eating for a specific period of time but instead just reduced caloric intake, it will not be discussed further in this report.

Protocols for IF and TRE are gaining popularity as weight loss programs, health benefits, and interventions in chronic diseases (Figure 1). They are suggested to help individuals achieve a clinically significant weight loss (>5% from baseline) (23), which would be relevant to improving cardiometabolic health biomarkers (24, 25). Some scholars consider IF and TRE different strategies (14). As such, TRE focuses on the timing of meals and their relation to circadian rhythm, hormonal, and metabolite profile within 24 h period, while IF does not affect the chronobiology as it does not restrict feeding during the feasting days (14, 26, 27).

There are also religious, ethical, and spiritual practices of fasting. For example, there are fasting practices in Judaism and Christianity. For example, fasting (abstaining from food) on Yom Kippur includes a full day from the previous day to the following evening; the orthodox Jews also abstain from drinking water for approximately 25 h (28). The literature also describes Ramadan fasting for practicing Muslims as an intermittent fasting strategy for weight loss; however, fasting in Ramadan is different than intermittent fasting as it requires a consecutive 29–30 days of fasting from sunrise to sunset with nothing per oral (NPO), including both food and beverages while IF, alternative day fasting, and 5/2 strategies allow for drinking water or zero calorie beverages or black coffee alternative during the fasting day (0–500 kcal per day). When designing diet strategies, however, it is crucial to consider the feasibility, adherence, and sustainability over time. If these strategies interfere with socialization, family time, dining on special occasions, cultural, ethnic, religious, or social eating patterns, they might not be sustainable or tolerable. These factors could affect weight loss maintenance to sustain the weight loss. Incorporating behavior modification and cognitive behavioral therapy strategies such as goal setting, self-monitoring, problem-solving, and portion size control are also encouraged as adjuvants to TRE, IF, and FMD (29–31).

## Types of studies of intermittent fasting and time-restricted eating diets

### Preclinical studies

#### Intermittent fasting

Animal models have been used as a model for glucose management in diabetes [reviewed in (28)]. For example, Tikoo et al. (32) reported that alternative day fasting did not affect the body weight in Sprague-Dawley rats compared to the control but improved the laboratory values of creatinine, albumin, and albumin improved the elevated blood pressure, and reduced type 1 diabetes induced nephropathy. In mice, Liu and colleagues reported that alternative-day fasting improved glucose tolerance, restored the pancreatic islet of Langerhans autophagy response, and enhanced beta cell survival (33).

#### Time-restricted eating

*In vitro* studies showed that periodic fasting might protect from oxidative damage, decreasing tumor growth and proliferation (34, 35). In animal models, TRE was investigated in mice where food availability was regulated during the 24 h circadian rhythm. The mice gained less weight, had a better blood glucose profile, longer life span, healthier muscle, and better cardiometabolic health when food was available only during a shorter window compared to the energy-matched mice fed ad-lib throughout the day (36–38). Mitchel and colleagues conducted a study in genetically homogenous male [*n* = 292 C57BL/6J (B6) mice comparing feeding ad libitum (AL) to either a 30% calorie restriction diet (CR) or single-meal feeding diet (MF)]. In the MF diet (caloric intake matched to the AL diet), the mice consumed the meal quickly, inducing self-imposed fasting (21 h), which led to significant improvement in health and decreased mortality rate as evidenced by the Kaplan-Meier survival analysis. Using a multiple comparison procedure, the main lifespan extension was 11% in MF and 28% in the CR group compared to the AL diet (39).

It is important to note that mice are nocturnal animals, and active eating time is at night. Similarly, studies in Drosophila showed that caloric restriction increased the lifespan and decreased age-related deterioration in cardiac function (40). The underpinning mechanisms of these findings involved several signaling pathways. Such pathways include mTOR (mechanistic Target of Rapamycin), which regulates autophagy (auto cell degradation for recycling of nutrients) and energy metabolic pathways (41), also AMPK (adenosine monophosphate kinase) and the circadian clock downstream targets.

Furthermore, mice fed a high-fat diet restricted to the active phase (in the dark) reduced fat mass and body weight and improved glycemic index compared to mice fed the same calories *ad libitum* (38).

Taken together, Findings from both IF and TRE suggest a beneficial effect of time-restricted feeding in murine models. However, the limitations in the translation of animal studies to humans lay in their different feeding patterns, where mice are nocturnal feeders. In contrast, human eats mainly during the day and under photopic light. Also, the duration of studies in animal models and humans varies significantly. Nevertheless, the animal model studies provided a proof-of-concept to be confirmed in human studies where the caloric intake was matched in Time-restricted feedings compared to the control subject fed ad lib (42, 43).

### Clinical studies

#### Intermittent fasting

Randomized controlled trials in human suggested that the weight loss associated with intermittent fasting are due to a reduction in calories and energy intake range between 10–30% compared to baseline (44–46). It has been shown to effectively achieve clinically significant weight loss (i.e., reduction of 5% body weight) in the short term. Strategies for intermittent fasting include a period of fasting alternating with feeding. Alternate Day Fasting (ADF) includes fasting for 1 day (0–500 kcal/day), followed by *ad libitum* eating for a day (Figure 1). During the fasting days, people could consume water or zero-calorie beverages. The other common type is the 5:2 diet which includes 5 days of eating *ad libitum* followed by 2 days of fasting. The 2 days could be either consecutive or separate days within a week. Although an individual could overeat during feasting days, there is a net deficit due to the alternating fasting days. However, it could be argued that adherence to and compliance with fasting days could be challenging to sustain and implement long-term.

#### Time restricted eating

Investigators tried various timed feeding to optimize the timing for maximal health outcome benefits. The clinical trials included early TRF from 8 AM to 5 PM, mid-TRE ending at 7 PM, or late TRE beginning at 12 PM. In a robust supervised randomized controlled crossover feeding study, Sutton et al. showed that timed feeding for 6 h (8 AM−2 PM) with the intake of 33% of the individual's daily energy requirements containing 50% carbohydrates (3 meals/day), improved insulin sensitivity, beta cell functions, and metabolic health, compared to the control group which was allowed to consume the same calories (33% daily energy requirements, 50% carbohydrates) for 12 h (8 AM-8 PM) tailored to the individual preferences (43). However, the study was conducted for 5 weeks in only 8 individuals who completed the study from 938 assessed for initial eligibility due to difficulty in recruitment for a supervised eating study. Similar findings were obtained from other short-term studies (26, 47), confirming the role of circadian glucose homeostasis rhythm in the cardiometabolic benefits of TRE. However, studies conducted in free-living conditions have altered both the caloric intake and timing of meals making it challenging to differentiate the independent effect of timing of food intake vs. caloric density's impact on metabolic health.

Furthermore, many studies did not quantify the dietary intake or discretionary calories or only provided baseline and final intervention data without dietary analysis (48–55). On the other hand, systematic reviews and meta-analyses of randomized controlled trials in patients with obesity found that fasting for 2–3 days (5:2 diet) led to lower body fat content and more weight loss than the matched controls who only had caloric restriction without time restriction (13, 56). However, studies with ADF and TRE, which restrict timed eating within 24 h. did not reveal similar findings (51, 57). Based on the current evidence, it appears that both caloric restriction and timing of meals are critical to weight loss and that time-restricted feeding has an independent beneficial effect on weight loss and improving insulin sensitivity in individuals with prediabetes (43).

## System biology benefits of IF and TRE

### Weight management

Studies have shown that IF and TRE have cardiometabolic benefits aligned with the circadian rhythm, independent of their weight loss outcomes. In a symposium review (58), Cienfuegos and colleagues attributed the cardiometabolic benefits of TRE to circadian rhythm and biological clocks, which affected glucose regulation, beta cell responsiveness, body composition, and body composition body weight, reduction of oxidative stress and metabolic switch (24, 59–62). As such, since fasting deprives the body of glucose, and after glycogen stores are depleted (within 12 h of fasting), the energy production is shifted to alternative energy sources such as fatty acids and ketone bodies, leading to the metabolic switch flipping (63). In addition, studies have shown that IF improves glucose regulation and decrease inflammatory biomarkers in the blood (64). Other mechanisms include augmented autophagy due to the downregulation of mTOR because the caloric restriction activates autophagy to eliminate damaged cellular content and recycle healthy cellular components (65, 66). IF studies, including ADF (45, 67–71) and the 5:2 diet (4, 72) studies found a 4–8% weight loss compared to the control in the short term. However, Long-term studies are required to directly compare the health benefits of IF and TRE in the same population for the same duration compared to the control group. More research would be warranted to address the sustainability and maintenance of weight loss and the metabolic differences between IF and TRE.

### Cardiometabolic benefits

Several clinical trials provided evidence that IF improved cardiometabolic biomarkers such as reducing LDL-C, BP, TG, insulin resistance, and HbA1C (24, 64, 73). Also, with IF protocols, people with insulin resistance and pre-diabetes have improved metabolic profiles and have lost weight (56). Further, studies have shown in T1D (74) and T2D (75) that IF is also effective in weight loss. However, other investigators reported that the time restriction benefits are not superior to the caloric restriction in the cardiometabolic outcome (44, 51, 76, 77).

### Inflammation and oxidative stress

Most clinical trials to date that measured the markers of inflammation, such as IL-6, TNF, and C-Reactive protein, did not show an effect of IF either ADF (57, 71) or 5:2 diet (78) or TRE (43, 79–81) on the inflammatory markers. However, combined TRE and exercise have been shown to improve the inflammatory markers, possibly due to the exercise component (82). On the other hand, RCTs have shown that IF (ADF, 5:2 diet) and TRE reduce the oxidative stress markers (4, 43, 79, 83), which might explain the improved insulin sensitivity (61, 62).

### The microbiome abundance and composition

IF and TRE can alter the microbiome composition and the host circadian rhythm or biological clock, thereby improving cardiometabolic health (15). However, individual variations of the host-microbe interactions may alter the host's response to diet. These variations may include differences in the microbial diversity, short-chain fatty acid (SCFA) composition, diversity of the microbiota, species abundance, phylum level, ratio of Firmicutes/Bacteroidetes, and microbial and host metabolic functions, which are altered in individuals with cardiometabolic diseases and may serve as discriminatory or predictive biomarkers (84, 85). It is also postulated that the gut microbiome modulates the energy metabolism diurnal rhythm by regulating the genes that control lipid uptake and adipose tissue thermogenesis during timed eating (86). Also, the microbiome can produce SCFA and ketone bodies that may be used as an energy source during fasting (87). Further, the feeding-fasting cycles affect the metabolic pathways, metabolites, and microbiome composition, which may drive the cardiometabolic advantage of IF and TRE. Other benefits of the IF on the microbiome include a possible role in reducing microvascular and macrovascular complications of diabetes (88) and brain health, as shown in animal models (89). However, more research is needed to confirm these findings in humans.

### Cancer and time-restricted diets

Tumor cells require nutrients and oxygen to grow and proliferate. Diet has been shown to play a role in tumor initiation and progression (90, 91). As such, diet modification can restrict nutrient availability to the tumor microenvironment or alter the tumor's metabolic vulnerabilities. Therefore, timed feeding may be targeted for inhibiting tumor growth or as an adjuvant for anticancer drugs (92). Studies in preclinical models show that nutrients play a role in tumor initiation, progression, cancer metabolism and survival (90, 93, 94). Moreover, recent findings using IF, TRE and FMD suggest that dietary interventions may be utilized as adjuvants to cancer therapy (19, 90, 92, 95, 96). Like personalized medicine, achieving precision nutrition mandates the provision of the right nutrients to the right patient at the right time. To do so, future studies need to analyze the tumor utilization of nutrients and the biochemical processes involved in the digestion and absorption of macronutrients and micronutrients.

## Potential side effects and contradictions

The contraindications for IF and TRE are like contradictions to dieting for weight loss in general. For example, IF and TRE are contraindicated in people with eating disorders or underweight (below 18.5 kg/m^2^). Also, IF and TRE are not recommended for pregnant women or lactating women and children under 12 years old (73). Clinicians should assess and monitor individuals for adverse effects frequency. When IF protocols are started, researchers should allow an adjustment period to allow participants to adapt to the protocol. For example, headache is reported, possibly due to dehydration, but subsides with increasing water intake (73). There was speculation about susceptibility to eating disorders, but evaluation of clinical trials of ADF and TRE did not show evidence of binge eating or purging behavior (97, 98). Also, there is no evidence of changed thyroid hormone levels (82), but the resting metabolic rate might be decreased with the 5–7% weight reduction following IF (57, 99). However, more research is needed to evaluate IF, FMD, and TRE's effects on health outcomes and the possible side effects.

## Challenges and limitations

Although the supervised TRE clinical trials documented the dietary caloric intake in the treatment and control groups, many free-living studies focused on the timing of meals without a complete characterization of the dietary intake in terms of caloric density and macronutrient intake. Additionally, many studies have not reported the body composition analysis, macronutrient distribution between meals, and numbers of meals and snacks within the timed-eating window; these limitations hinder comparing the time-restricted eating strategies with the traditional caloric-restriction standardized weight loss programs that account for total caloric intake (Figure 1). Furthermore, most studies to date were short-term, ranging between 8–12 weeks; thus, the long-term impact of the IF and TRE has not been thoroughly studied and is primarily undetermined (100). Moreover, the long-term success of weight loss and weight loss maintenance is not known yet and remains to be seen. Importantly, future studies should expand to include different populations, various ethnic groups, various personal dietary preferences, and discrepancies in individual responses to IF and determine the feasibility of such protocols, accessibility of meals, and long-term adherence to the diet. The assumption is that since people have less time to eat, they will eat fewer calories as it is hard to fit a large volume in a short period of time; however, this has not been confirmed by empirical data.

## Recommendations

It would be informative to document the dietary composition, meal composition, intake of discretionary calories, and caloric density in addition to the timing of meals to differentiate the effects of timing of feeding vs. caloric restriction, diet composition, or macronutrient intake. These factors (caloric restriction and time restriction) could work synergistically or with an additive effect to improve metabolic health outcomes. For example, 3-consecutive days' diet records, including 2 weekdays and 1 weekend, should be analyzed throughout the studies. A more detailed analysis would help determine possible synergism between diet quality such as macronutrients, micronutrients, dietary pattern, food groups, supplements and herbs, the amount of processed food intake and the timing of meals and snacks. To translate the recent advances in the IF and TRE research into practical applications, it would be worthwhile to incorporate the diet quality of what we eat with insights from the timing of when we eat it. Since the goal of studying TRE and IF is to provide precision nutrition recommendations, future directions should include studying the best timing window and diet composition and quality of diet, including the intakes of whole grain, plant-based diet, limiting ultra-processed food, and portion control. Furthermore, the benefits of IF and TRE should be evaluated by RCTs in different populations, ethnic groups, ages, geographic differences, physical activity levels, body composition, BMI, and in patients with obesity, type 1 or type 2 diabetes, metabolic syndrome, and cardiovascular disease. It is possible that individualized, tailored IF and TRE protocols may be adopted for personalized precision nutrition that increases compliance, tolerability, and sustainability to achieve optimal health outcomes.

## Data availability statement

The original contributions presented in the study are included in the article/supplementary files, further inquiries can be directed to the corresponding author.

## Author contributions

GS researched, reviewed the literature, wrote the paper, and prepared the figure.

## Conflict of interest

The author declares that the research was conducted in the absence of any commercial or financial relationships that could be construed as a potential conflict of interest.

## Publisher's note

All claims expressed in this article are solely those of the authors and do not necessarily represent those of their affiliated organizations, or those of the publisher, the editors and the reviewers. Any product that may be evaluated in this article, or claim that may be made by its manufacturer, is not guaranteed or endorsed by the publisher.
